# Metagenomic surveillance reveals off-season circulation of respiratory viruses during the COVID-19 pandemic in Salvador, Brazil

**DOI:** 10.1016/j.nmni.2026.101717

**Published:** 2026-02-06

**Authors:** Juan P. Aguilar Ticona, Luciane Amorim Santos, Xiao Meng, Nivison Nery, Mariam O. Fofana, Laise de Moraes, Icaro Morais Strobel, Renato Vitoriano, Marina Silveira Cucco, Emilia M.M. Andrade Belitardo, Gowtham Thakku, Jaqueline S. Cruz, Angela M. Detweiler, Norma Neff, Cristina M. Tato, Mitermayer G. Reis, Federico Costa, Derek A.T. Cummings, Albert I. Ko, Ricardo Khouri

**Affiliations:** a Instituto de Saúde Coletiva, Universidade Federal da Bahia, Salvador, BA, Brazil; b Instituto Gonçalo Moniz, Fundação Oswaldo Cruz, Ministério da Saúde, Salvador, BA, Brazil; c Department of Epidemiology of Microbial Diseases, Yale School of Public Health, New Haven, CT, United States; d Programa de Pós-Graduação em Microbiologia, Universidade Federal da Bahia, Salvador, BA, Brazil; e Department of Laboratory Medicine, Peking Union Medical College Hospital, Beijing, China; f Department of Emergency Medicine, Emory University School of Medicine, Atlanta, GA, United States; g Instituto de Ciências da Saúde, Universidade Federal da Bahia, Salvador, BA, Brazil; h Chan Zuckerberg Biohub, San Francisco, California, United States; i Faculdade de Medicina da Bahia, Universidade Federal da Bahia, Salvador, BA, Brazil; j Department of Biology, University of Florida, Gainesville, FL, United States; k Emerging Pathogens Institute, University of Florida, Gainesville, FL, United States; l Department of Epidemiology, Johns Hopkins Bloomberg School of Public Health, Baltimore, MD, United States; m Department of Biomedical Engineering, Johns Hopkins Whiting School of Engineering, Baltimore, MD, United States; n Rega Institute for Medical Research, Katholieke Universiteit Leuven, Leuven, Belgium

**Keywords:** Respiratory viruses, Metagenomics, COVID-19, Pandemic

## Abstract

**Background:**

Evidence from multiple countries suggests that the COVID-19 pandemic disrupted the transmission of other respiratory viruses. We characterized respiratory virus transmission during the pandemic in Salvador, Brazil, a tropical region in the Southern Hemisphere.

**Methods:**

From November 2021 to October 2022, we conducted biweekly household visits in an urban informal settlement to screen individuals with respiratory symptoms. Symptomatic individuals and their contacts were interviewed, and nasal swabs collected. Virus identification was performed using multiplex RT-qPCR, followed by metagenomic analysis in a subset of symptomatic participants with negative RT-qPCR results.

**Results:**

We screened 3174 residents from 1174 households, identifying 669 symptomatic episodes and detecting 219 respiratory viruses. including coinfections, SARS-CoV-2 was the most common with 118 cases (54%), followed by Influenza A with 39 (18%), Rhinovirus with 22 (10%), Human Parainfluenza Virus with 15 (7%), Respiratory Syncytial Virus with 13 (6%), and seasonal Human Coronaviruses with 12 (5%). Co-infections were observed, with combinations involving SARS-CoV-2, Influenza A, and Respiratory Syncytial Virus being the most common. Peaks of Influenza, HPIV, and RSV occurred in late 2021 during low Delta circulation, while Omicron BA.1 emerged in January 2022. Influenza and RSV showed low transmission during Brazil's winter months, and seasonal coronaviruses reappeared two years after the pandemic onset.

**Conclusion:**

Multiplex RT-qPCR and metagenomic analysis allowed rapid detection and sequencing. An off-season influenza peak was identified, possibly due to relaxed hygiene measures or accumulated susceptibility after SARS-CoV-2 interventions. The household secondary attack rate for influenza was lower than for Omicron BA.1, possibly reflecting lower transmissibility or pre-existing immunity.

## Highlights

• We identified an off-season influenza peak during the summer, following low transmission throughout the year.
• We identified the circulation of HCoV after 2 years of COVID-19 pandemic began.
• We implemented a metagenomic analysis for simultaneous detection of multiple respiratory viruses.
• Our results highlight the value of metagenomic surveillance to detect unexpected viral circulation in tropical regions.

## 1 Introduction

Before the COVID-19 pandemic, respiratory viruses such as influenza, respiratory syncytial virus (RSV), and non-SARS coronaviruses typically exhibited seasonal peaks during the Brazilian winter [1]. This pattern, however, was disrupted during the pandemic, with lower incidence during the usual season and unexpected increases during off-seasons [[1], [2], [3], [4]]. Multiple factors have been proposed to explain these shifts, including non-pharmaceutical aimed at preventing the spread of Severe Acute Respiratory Syndrome Coronavirus 2 (SARS-CoV-2) transmission and potential immunological interactions between SARS-CoV-2 and other respiratory pathogens [5,6]. In the US, early in the pandemic, cases of both influenza and bacterial pneumonia declined, largely due to these preventive measures [7]. However, not all viruses followed this trend—rhinovirus, for instance, saw increased circulation in Japan, likely due to virus-specific characteristics [8]. Respiratory virus infections occurring during non-typical seasons can significantly impact public health services, increasing hospitalizations and higher demand for healthcare resources.

During the pandemic, broad testing for respiratory viruses in patients under investigation for SARS-CoV-2 was inconsistent, hindering a comprehensive understanding of alternative viral infections and coinfections [9]. While the epidemiology of influenza and RSV is well-documented, data on less common respiratory viruses and their interaction with SARS-CoV-2 remain limited [10]. Seasonal human coronaviruses, for example, declined markedly, but information on their circulation during this period is still limited [11,12]. Available data mostly stems from passive national surveillance, which may not capture the true burden due to the prioritization of COVID-19 response efforts. Therefore, the objective of our study was to characterize respiratory virus dynamics and co-infections in a tropical setting using metagenomic tools, with particular emphasis on the re-emergence of respiratory viruses observed two years after the onset of the COVID-19 pandemic. To address this aim, we conducted active case finding in an urban informal settlement in Salvador, Brazil, identifying symptomatic respiratory cases during the pandemic. This approach revealed the local transmission of the SARS-CoV-2 BA.1 variant in early 2022 [13]. We describe the incidence of other respiratory viruses during the reduction of cases associated with the declining phase of transmission of SARS-CoV-2 Delta variant and the emergence of the Omicron variant. We also characterize the performance of metagenomic analysis, including either Shotgun approach (metagenomic sequencing without prior enrichment) or Hybrid-capture (target enrichment using probe-based capture) method, to detect and sequence the whole genome of different respiratory viruses.

## 2 Methods

### 2.1 Study design, setting and participants

**Fig. 1:**
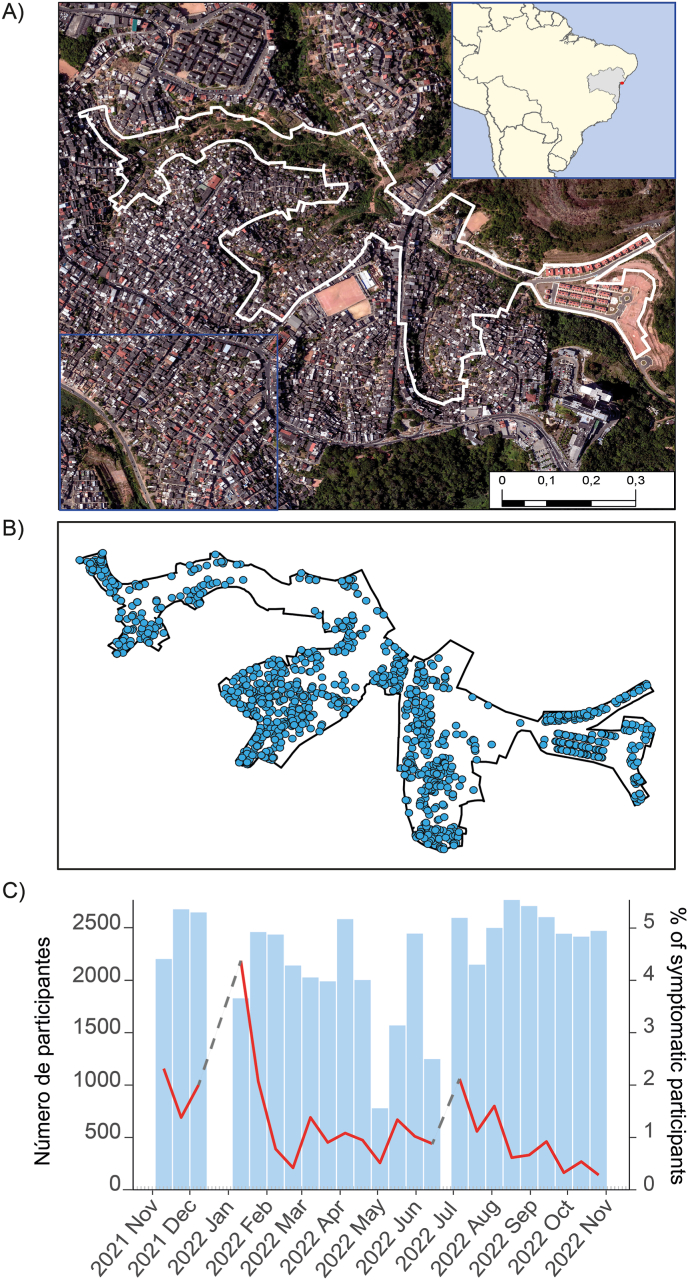
Study site and participants, A) Aerial image of the study area, with insets depicting the location of the study area within Brazil and Bahia, and a photo of the study area; B) Household included in the active case finding; and C) Number participants followed. Blue bars represent the number of participants followed in each biweekly visit, the red line shows the percentage of symptomatic individuals and the dashed gray line represents the break for holidays.

We developed an active case-finding study nested in an ongoing cohort living in the urban informal settlement of Pau da Lima, Salvador, Brazil (Fig. 1A). The main characteristics of this community have been previously described [13]. In 2021, we conducted a census identifying 3364 inhabitants residing in 1174 households in the study area (0.35 km^2^) (Fig. 1B). Like many urban informal settlements, this community grapples with poverty, insufficient sanitation facilities, and the absence of legal home ownership titles. In this area, a previous study using data from 2009 to 2013 identified that influenza-like illness cases had an annual cyclical component, with peaks typically occurring in the months of August and September and with a mean annual incidence of 60 cases/1000 persons per year [14].

### 2.2 Recruitment

From November 2021 to October 2022 (after the emergence of SARS-CoV-2 and the subsequent decline in respiratory cases; Supplementary Fig. 1), a trained field team conducted biweekly household visits in the study area to identify residents with respiratory or SARS-CoV-2–associated symptoms [13]. Using a standardized questionnaire, they recorded cases presenting symptoms such as fever, cough, fatigue, headache, myalgia, sore throat, coryza, dyspnea, anorexia, nausea/vomiting, diarrhea, or altered mental status. When symptomatic individuals were identified, the team conducted interviews to collect sociodemographic data and information on symptom onset and duration. Anterior nasal swabs were collected from all household members if any were symptomatic. A follow-up visit was scheduled seven days later to identify new symptomatic cases and collect additional samples. Participants unavailable during visits were rescheduled within the same biweekly round.

### 2.3 Molecular diagnosis protocol

The laboratory diagnostic protocol consisted of two stages. First, all samples were tested for SARS-CoV-2 using RT-qPCR with one of the following kits: Biomol OneStep/COVID-19 (Instituto de Biologia Molecular do Paraná), CDC 2019-nCoV RT-PCR Assay, or Molecular SARS-CoV-2 Kit EDx (Bio-Manguinhos). Additionally, a multiplex RT-qPCR assay (Allplex Seegene, Seoul, South Korea) targeting Influenza A, Influenza B, RSV, and SARS-CoV-2 (3 genes) was used to validate SARS-CoV-2 results and expand detection to other respiratory viruses. SARS-CoV-2–positive samples were then submitted for whole genome sequencing using the Illumina COVIDSeq Test for lineage determination.

In the second stage, metagenomic analysis was performed on samples positive for Influenza A, B, or RSV to identify genotypes, and on RT-qPCR–negative samples from individuals with fever or cough to detect other respiratory pathogens. Two approaches were applied: an untargeted Shotgun metagenomics method and a targeted Hybrid-capture method using the Illumina Respiratory Virus Oligo Panel (RVOP). While the Shotgun approach enables broad pathogen detection, RVOP offers a cost-effective and sensitive alternative for sequencing over 40 common respiratory viruses. Both methods support whole genome sequencing. Pathogen identification and genome assembly were conducted using the CZID platform (https://czid.org/). Influenza genome assembly was performed using the ViGEAS workflow (https://github.com/khourious/vigeas) see Supplementary Methods for details.

### 2.4 Data analysis

We utilized R version 4.2.2 software (https://www.r-project.org) for data analysis. The prevalence of each respiratory virus, as well as the SARS-CoV-2 variants and subvariants, were summarized in tables using frequency and percentage. We compared the symptomatology of COVID-19 cases and the most prevalent respiratory virus infections with symptomatic participants without a defined etiology in the community, using chi-square tests to compare the presence of symptoms and the *U* test to compare the number of symptoms by duration. The symptom profiles of coinfections were also compared with those of the most prevalent monoinfections. To measure associations, odds ratios (ORs) and 95% confidence intervals (CIs) were calculated. Subsequently, generalized linear models were performed to evaluate factors associated with the most prevalent infections, with variable selection conducted using backward elimination methods. Participants with missing data were excluded from the analyses.

For calculating the clinical secondary attack rate (SAR), defined as the proportion of household contacts who developed symptoms and became clinical secondary cases during the 14-day follow-up period, we assumed that the earliest positive respiratory virus test or symptom onset in a household represented the index case. Co-index cases were considered if multiple household members tested positive or exhibited symptoms on the same date. In this case, to compute the SAR, one co-index case was randomly selected. Household contacts were individuals residing with the index cases during the 7 days post-positive RT-qPCR test or symptom onset. A secondary case referred to someone testing positive for SARS-CoV-2 among household contacts. The SAR was determined by dividing the number of secondary cases by the total number of non-index household residents.

## 3 Results

**Fig. 2:**
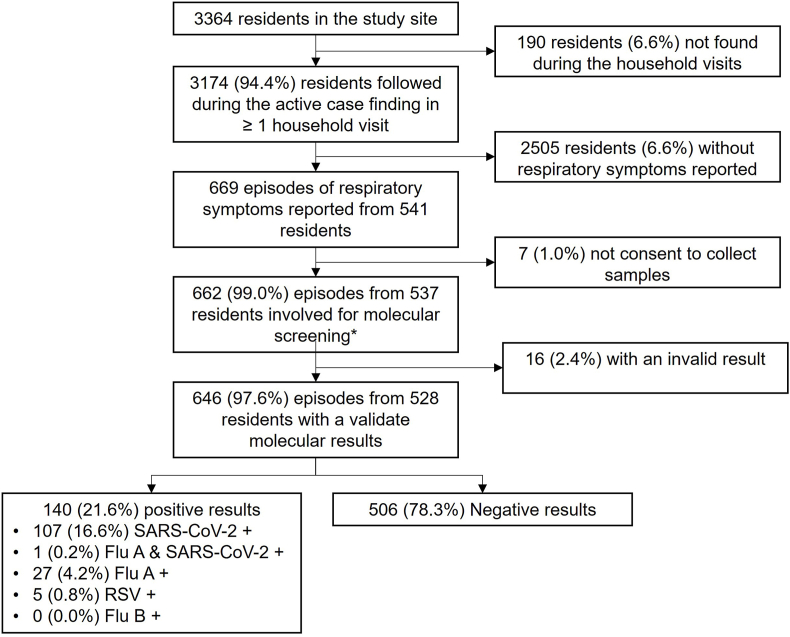
Study flowchart.

**Fig. 3:**
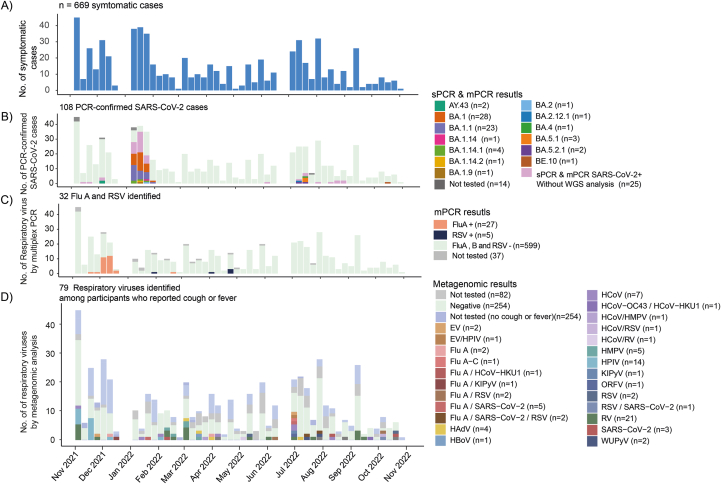
Respiratory virus identification. A) Symptomatic cases identified during the active case finding (n = 669); B) SARS-CoV-2 confirmed by single RT-qPCR and multiplex RT-qPCR and variants identified by the Whole Genomic Sequencing analysis (n = 108); C) Flu and RSV detection by the multiplex RT-qPCR (n = 32); D) Respiratory virus identified by metagenomic among participants who reported cough or fever during the visits (n = 79; and the frequencies and subcategories are included in Supplementary Table 3). EV – Enterovirus; Flu A – Influenza A virus; Flu A–C – Influenza A, B or C virus; HCoV-HKU1 – Human coronavirus HKU1; HMPV – Human metapneumovirus; HPIV – Human parainfluenza virus; KIPyV – KI polyomavirus; ORFV – Orf virus; RSV – Respiratory syncytial virus; RV – Rhinovirus; SARS-CoV-2 – Severe acute respiratory syndrome coronavirus 2; WUPyV – WU polyomavirus; HAdV – Human adenovirus; HBoV – Human bocavirus.

We conducted 24 rounds of biweekly household visits, successfully engaging 1098 out of 1174 households (94%) in at least one visit, involving a total of 3174 residents (Supplementary Table 1). Among these residents, 541 reported experiencing at least one symptomatic episode, resulting in a total of 669 reported symptomatic respiratory episodes between November 2021 and October 2022 (Fig. 1, Fig. 2, Fig. 3A). Of the 541 participants, 349 (65%) were female, with a median age of 30 years (IQR: 15–44).

A total of 219 viruses were identified throughout the study, including 118 (54%) cases of SARS-CoV-2, 39 (18%) of Influenza A, 22 (10%) of Rhinovirus, 15 (7%) of Human Parainfluenza,13 (6%) of RSV, and 12 (5%) of Human Coronavirus. These numbers include co-infections and are represented in Fig. 3. Other viruses with a frequency lower than 5 (2%) are detailed in Supplementary File 1 and Supplementary Table 2 The following paragraphs sequentially present the findings of our pathogen identification protocol using PCR and metagenomics.

In the first stage of the study, utilizing both single and multiplex RT-qPCR, we identified 107 cases of SARS-CoV-2 among symptomatic participants residing in 76 households (Fig. 3B). Subsequently, we performed Whole Genome Sequencing (WGS) on 69 cases, of which 57 (82.6%) achieved >90% coverage. These sequences were then submitted to the GISAID repository (Supplementary File 2). Most of these cases were associated with the Omicron BA.1. and BA.1.1 variants, which were predominant during the first trimester of 2022. Only 2 (3.5%) cases of the Delta variant were identified, both in November 2021 (Fig. 3B and Supplementary Table 3). Furthermore, multiplex RT-qPCR identified 27 cases of Influenza A, one coinfection between Influenza A and SARS-CoV-2 and 5 cases of RSV; no cases of influenza B were identified. Influenza A predominated in November and December 2021, during the low transmission of the Delta variant and preceding the increase of Omicron BA.1∗ cases (Fig. 3C). After November and December, Influenza A and RSV, did not show any increase in cases, even during the winter season (June-August), which are typically the circulating season for these two pathogens (Fig. 3C).

In the second stage of our testing, among 415 symptomatic participants experiencing fever and cough, who initially tested negative in the first stage described above, we detected the presence of 21 (5%) human rhinovirus and 14 (4%) Human Parainfluenza Virus (HPIV) cases (not accounting for coinfections), which were the most frequent in November and December 2021, along with other pathogenic agents (see Supplementary Table 3 and Fig. 1D) identified by metagenomic methods. In addition to the RT-qPCR tests used to detect SARS-CoV-2, RSV, and Influenza, the metagenomic analysis identified 2 additional cases (0.4%) of RSV, 2 cases (0.4%) of SARS-CoV-2 and 2 cases (0.4%) of Influenza A.

In addition to the Influenza A virus and SARS-CoV-2 co-infection identified in the first stage, the metagenomic analysis revealed additional 18 co-infection cases among the 415 individuals analyzed (3.9%). The most frequent combination was Influenza A and SARS-CoV-2, with 5 cases (1.2%), followed by Influenza A, SARS-CoV-2, and RSV with 2 cases (0.5%), and Influenza A and RSV, also with 2 cases (0.5%). Other detected co-infections included RSV and SARS-CoV-2 in 1 case (0.2%), as well as combinations of different human coronaviruses, such as HCoV-HKU1 and HCoV-OC43 in 1 case (0.2%). Additionally, co-infections with other respiratory viruses were identified, including HAdV-C/HMPV, HCoV-229E/HMPV, HCoV-HKU1/RV-C, EV-B/HPIV-2, HCoV-OC43/RSV and Flu A/HCoV-HKU1, each with 1 case (0.2%), see Supplementary Table 2 for details. Symptoms associated with each coinfection are summarized in Supplementary Table 3. The comparison between the most prevalent coinfection, Influenza A and SARS-CoV-2, and the corresponding monoinfections is presented in Supplementary Table 4. Notably, only headache was observed at a lower frequency in coinfections compared with Influenza A monoinfections (2 of 6 Influenza A and SARS-CoV-2, 33.3% vs. 23 of 29 Influenza A, 79.3%; *p* = 0.043). Finally, the genomic sequence was completed in 17 associated with the H3N2 genotype. However, it's noteworthy that 329 (79.7%) participants were not fully tested using metagenomic techniques, rendering the evaluation incomplete throughout the study year (Supplementary File 1).

Of 48 samples tested by RT-qPCR and Shotgun, Shotgun detected SARS-CoV-2 and RSV in one sample each, missed two Influenza A cases, and found other viruses in nine RT-qPCR-negative samples. The remaining 35 samples, including 10 healthy controls, were negative by both methods. Among 332 samples tested by RT-qPCR and Hybrid-capture, Hybrid-capture identified 33 Influenza A-positive cases, 11 of which were RT-qPCR negative. For SARS-CoV-2, one case was concordant, and seven were detected only by Hybrid-capture. Additionally, Hybrid-capture identified RSV in eight RT-qPCR-negative samples and detected other viruses in 57 samples not covered by the RT-qPCR panel. A total of 239 samples were negative by both methods. Hybrid-capture showed the highest detection capacity, identifying Influenza A virus cases missed by Shotgun but included in the RT-qPCR panel. It also aligned with Shotgun in detecting HPIV-4, which was not targeted by RT-qPCR. One sample tested negative by all three methods (Supplementary File 1 and Supplementary Fig. 2).

Each method—RT-qPCR, metagenomic Shotgun sequencing, and Hybrid-capture—exhibited distinct detection profiles. RT-qPCR provided high specificity but was constrained by its limited target panel. Shotgun sequencing enabled the identification of both viral and non-viral respiratory pathogens, such as *Haemophilus influenzae*, *Moraxella catarrhalis*, and *Streptococcus pneumoniae*, although its sensitivity for certain viruses, notably Influenza A, was comparatively lower. In contrast, the Hybrid-capture approach demonstrated the broadest detection capacity, identifying viral agents beyond the scope of both RT-qPCR and Shotgun methods. Regarding genomic contributions, 69 SARS-CoV-2 and 17 Influenza virus genomes were deposited in GISAID. Additionally, 33 complete or near-complete genomes from a diverse array of respiratory viruses—including Human metapneumovirus (HMPV), Human adenovirus B (HAdV-B), Human bocavirus (HBoV), KI polyomavirus (KIPyV), Rhinoviruses A, B, and C, Human parainfluenza viruses (HPIV) 1–4, Respiratory syncytial virus A (RSV-A), and seasonal coronaviruses 229E, HKU1, and OC43—were submitted to GenBank (Supplementary Table 5, Supplementary File 1).

Secondary transmission was assessed among Influenza A cases due to the completeness of testing in household participants during the study period and the high incidence. Among these cases, we identified 10 households with at least one Influenza A case and two or more residents. A total of 30 participants were identified, including 10 index cases and 20 contacts. Within the contact group, 5 secondary cases emerged, yielding a 25% (95% CI: 9.8 – 47.0) secondary attack rate. Notably, no asymptomatic secondary cases were detected.

Finally, we analyzed symptom frequency among participants infected with the most commonly detected viruses. Median ages for individuals with SARS-CoV-2, Influenza A, Rhinovirus, and RSV were comparable to those with negative results. However, participants with HPIV had a significantly lower median age of 10 years (IQR: 4.0–27.0) versus 30 years (IQR: 15.0–47.0) in the negative group (p = 0.006). The description of symptom frequencies and the comparison with cases without an identified etiology through the study protocol are presented in Table 1 and Supplementary Table 6. Participants with SARS-CoV-2 more frequently reported sore throat (p = 0.005), fever (p = 0.002), chills (p = 0.046), headache (p = 0.001), and myalgia (p = 0.035), while cough was more common in the negative group (p = 0.004). The SARS-CoV-2 group also had a higher total number of symptoms (p = 0.016). Among those with Influenza A, fever (p = 0.008), headache (p < 0.001), and myalgia (p < 0.001) were more frequent, whereas cough remained more common in the negative group (p = 0.016). Rhinovirus and RSV infections were both associated with a higher frequency of runny nose (p = 0.036 and p = 0.037, respectively). Notably, RSV infected participants exhibited fewer symptoms overall compared to the negative group (p = 0.007). Multivariable models identified the key clinical features associated with SARS-CoV-2 and Influenza A infection. For SARS-CoV-2, headache (OR = 1.67, 95% CI: 1.04–2.68, p = 0.033) and the number of respiratory symptoms (OR = 1.52, 95% CI: 1.16–2.01, p = 0.002) were positively associated, while cough was negatively associated (OR = 0.26, 95% CI: 0.13–0.53, p < 0.001). In contrast, Influenza A was strongly associated with headache (OR = 3.59, 95% CI: 1.44–10.24, p = 0.009), anorexia (OR = 4.76, 95% CI: 2.03–11.09, p < 0.001), and showed a marginal significance (OR = 1.57, CI: 0.98–2.50, p = 0.059) with fever, whereas cough showed a negative association (OR = 0.29, 95% CI: 0.11–0.77, p = 0.010). Analyses for other viruses were limited by the smaller number of detected cases (Supplementary Table 7).

**Table 1:** Frequency of symptoms among prevalent respiratory virus infections.

	SARS-CoV-2	FluA	Rinovirus	HPIV	RSV	Negative	SARS-CoV-2 vs Negative	FluA vs Negative	Rinovirus vs Negative	HPIV vs Negative	RSV vs Negative
	N = 109	N = 29	N = 21	N = 14	N = 7	N = 337	p-value	p-value	p-value	p-value	p-value
Age, median (IQR)	36.0 (22.0, 51.0)	31.0 (13.0, 38.0)	18.0 (13.0, 42.0)	10.0 (4.0, 27.0)	38.0 (17.0, 44.0)	31.0 (15.0, 47.0)	0.100	0.257	0.207	**0.005**	0.937
Sex, n (%)							0.731	0.842	0.819	1.000	1.000
Female	72 (66.1%)	18 (62.1%)	13 (61.9%)	9 (64.3%)	5 (71.4%)	216 (64.1%)					
Male	37 (33.9%)	11 (37.9%)	8 (38.1%)	5 (35.7%)	2 (28.6%)	121 (35.9%)					
Symptoms											
Cough	84 (77.1%)	21 (72.4%)	18 (85.7%)	12 (85.7%)	4 (57.1%)	300 (89.0%)	**0.004**	**0.016**	0.717	0.660	**0.037**
Runny nose	70 (64.2%)	22 (75.9%)	18 (85.7%)	9 (64.3%)	5 (71.4%)	212 (62.9%)	0.820	0.226	**0.036**	1.000	1.000
Sore throat	61 (56.0%)	8 (27.6%)	10 (47.6%)	8 (57.1%)	1 (14.3%)	136 (40.4%)	**0.005**	0.235	0.504	0.269	0.251
Shortness of breath	16 (14.7%)	2 (6.90%)	4 (19.0%)	2 (14.3%)	1 (14.3%)	50 (14.8%)	1.000	0.403	0.538	1.000	1.000
Fever	57 (52.3%)	18 (62.1%)	6 (28.6%)	6 (42.9%)	0 (0%)	120 (35.6%)	**0.002**	**0.008**	0.640	0.580	0.101
Chills	23 (21.1%)	5 (17.2%)	2 (9.52%)	2 (14.3%)	0 (0%)	44 (13.1%)	**0.046**	0.567	1.000	0.704	0.602
Headache	69 (63.3%)	23 (79.3%)	9 (42.9%)	10 (71.4%)	1 (14.3%)	152 (45.1%)	**0.001**	**<0.001**	1.000	0.060	0.137
Loss taste	9 (8.26%)	4 (13.8%)	3 (14.3%)	0 (0%)	0 (0%)	26 (7.72%)	0.839	0.280	0.236	0.611	1.000
Loss smell	6 (5.50%)	2 (6.90%)	4 (19.0%)	0 (0%)	0 (0%)	24 (7.12%)	0.664	1.000	0.071	0.611	1.000
Fatigue	22 (20.2%)	8 (27.6%)	2 (9.52%)	2 (14.3%)	1 (14.3%)	49 (14.5%)	0.176	0.103	0.751	1.000	1.000
Myalgia	25 (22.9%)	6 (20.7%)	3 (14.3%)	1 (7.14%)	0 (0%)	47 (13.9%)	**0.035**	0.405	1.000	0.702	0.600
Anorexia	14 (12.8%)	13 (44.8%)	2 (9.52%)	4 (28.6%)	0 (0%)	40 (11.9%)	0.866	**<0.001**	1.000	0.084	1.000
Nausea	15 (13.8%)	3 (10.3%)	1 (4.76%)	4 (28.6%)	0 (0%)	44 (13.1%)	0.871	1.000	0.494	0.109	0.602
Diarrhea	14 (12.8%)	3 (10.3%)	3 (14.3%)	3 (21.4%)	0 (0%)	38 (11.3%)	0.731	1.000	0.720	0.217	1.000
Altered mental state	2 (1.83%)	0 (0%)	0 (0%)	0 (0%)	0 (0%)	2 (0.59%)	0.252	1.000	1.000	1.000	1.000
No. of symptoms, median (IQR)	4.0 (3.0, 6.0)	4.5 (2.5, 6.5)	3.0 (2.0, 5.0)	5.0 (4.0, 5.0)	2.0 (1.0, 3.0)	3.0 (2.0, 5.0)	**0.016**	0.052	0.863	0.067	**0.007**
No. of respiratory symptoms, median (IQR)	2.0 (1.0, 3.0)	2.0 (1.0, 2.0)	2.0 (2.0, 3.0)	2.0 (2.0, 3.0)	2.0 (1.0, 3.0)	2.0 (1.0, 3.0)	0.160	0.697	0.058	0.880	0.717

SARS-CoV-2, Severe Acute Respiratory Syndrome Coronavirus 2; Flu A, Influenza A virus; HPIV, Human Parainfluenza Virus; RSV, Respiratory Syncytial Virus; IQR, Interquartile Range.

## 4 Discussion

This year-long longitudinal study in a community cohort enabled a comprehensive characterization of respiratory virus dynamics and diversity amid high viral circulation two years after the onset of the COVID-19 pandemic. We identified off-season transmission of Influenza A in an urban informal community in a tropical region during November and December 2021, coinciding with low circulation of the SARS-CoV-2 Delta variant and preceding the emergence of the Omicron BA.1∗ variant. We also observed the re-emergence of other seasonal human coronaviruses (HCoV-229E, HCoV-HKU1, and HCoV-OC43) and additional respiratory viruses, reflecting the gradual restoration of viral circulation after COVID-19 disruptions. Furthermore, we described the symptoms associated with specific infections. Finally, we identified a low proportion of coinfections, which are not commonly reported, highlighting viral interference among respiratory pathogens. [15]. The combination of conventional molecular techniques (RT-qPCR) with metagenomic approach provided a comprehensive view of respiratory pathogen dynamics. The successful implementation of this protocol in an urban informal setting demonstrates its feasibility in resource-limited environments, highlights the value of active community-based surveillance, and underscores the need to extend such strategies to vulnerable populations that are often neglected. This approach not only enhances the understanding of respiratory virus epidemiology in tropical regions but also contributes to global preparedness for future respiratory virus outbreaks.

Following the COVID-19 emergency declaration and widespread preventive measures, influenza cases declined notably [5,16]. Although tropical regions typically show multiple annual peaks of respiratory virus activity, they also experienced a substantial drop in influenza during the pandemic. For example, Singapore, which usually has two influenza peaks (April–July and November–January) [17]. Influenza emergence often coincided with low SARS-CoV-2 incidence, as seen in several European countries where Influenza A and B resurged during periods of reduced COVID-19 transmission [18]. Similarly, in China, stringent COVID-19 measures initially suppressed Influenza B, but cases rose in 2021 following a decline in SARS-CoV-2 and before Omicron's emergence [19]. Correspondingly, surveillance data from December 2021 in Rio de Janeiro and São Paulo, Brazil, demonstrated an atypical off-season surge of Influenza A during the summer, diverging from the usual bimodal seasonal pattern with peaks in June and January. This atypical circulation illustrates how respiratory virus seasonality has shifted in the post-pandemic period and may extend into subsequent years. In 2023, passive surveillance in southern Brazil showed that SARS-CoV-2 remained the most frequently detected virus, followed by RSV and influenza, both peaking in the fall [20]. Recent evidence suggests that diminished population immunity could heighten the risk of broader and potentially more severe influenza epidemics as viral circulation returns [21,22]. This disruption, increasingly detected in several countries is manly associated to the relaxation of non-pharmaceutical interventions, but likely reflects diminished viral interference due to reduced SARS-CoV-2 circulation, coupled with circulation of the H3N2/Darwin strain, which was not included in the vaccine formulation for that season [1,3,15].

Seasonal human coronaviruses, alongside influenza, are among the leading causes of upper respiratory tract infections worldwide [23]. During the early COVID-19 pandemic, sustained SARS-CoV-2 transmission occurred with an apparent absence of seasonal coronaviruses [24]. However, substantial gaps in genomic and clinical surveillance (driven by limited sampling and underreporting) continue to hinder a full understanding of HCoV reemergence. Our study (in a tropical region and trough an active surveillance) provides an opportunity to characterize the return of HCoVs during the COVID-19 pandemic, before the BQ.1 and XBB waves, the last Omicron variants to cause significant SARS-CoV-2 transmission in Brazil [25]. Together with national surveillance data, our findings show that the early suppression of viral circulation temporarily altered HCoV dynamics, but this effect was not sustained, leading to an asynchronous reemergence alongside other respiratory viroses [12,26]. Surveillance and sequencing efforts like ours are crucial for monitoring the ongoing evolution of HCoVs—including the emergence of new lineages and recombination events, and for understanding how transmissibility, clinical presentation, and immunity durability vary across populations. This is particularly relevant in communities such as Pau da Lima, where (with high hybrid immunity at the time of our study) more than 50% of residents had received at least one SARS-CoV-2 vaccine dose and 76% were already seropositive two years into the pandemic [25]. Notably, during this period, seasonal coronaviruses (HCoV-229E, HCoV-HKU1, and HCoV-OC43) were again detected circulating in our study community. Similar trends were observed for RSV, which surged beyond national historical levels in pediatric populations [6,27]. Our study, limited to children over two years old, captured this resurgence primarily among older children and adults.

The detection of influenza, RSV, and seasonal human coronaviruses represents only a fraction of the respiratory viruses identified in our study. By combining RT-qPCR with metagenomic sequencing, we employed a comprehensive diagnostic approach that uncovered a broader spectrum of pathogens, including co-infections and underreported viruses, providing a more complete view of respiratory viral diversity and interactions in the community. Influenza A (Flu A) and RSV were the most frequent combinations of co-infections detected with SARS-CoV-2. Flu A co-infections have been reported since early in the pandemic, with a surge during the Delta-to-Omicron transition, a phenomenon colloquially termed “Flurona" [21]. Our findings are consistent with previous reports identifying RSV and influenza A as the most frequently co-infected viruses associated with SARS-CoV-2 [10], as well as with laboratory models showing that Influenza A (specifically the H3N2 strain identified in our study) induces viral interference against SARS-CoV-2 through an interferon (IFN) response [15]. These results underscore the need for broad-spectrum viral surveillance to monitor evolving respiratory viruses and their interactions.

Diagnosing respiratory virus infections remains challenging due to overlapping clinical symptoms across different viruses, complicating the clear definition of syndromes such as the common cold or influenza-like illness [23]. In our study, the most frequently detected viroses (SARS-CoV-2, Influenza A, Rhinovirus, Parainfluenza, and RSV) exhibited symptom profiles largely similar to those of symptomatic individuals who tested negative. Notably, cough was more common among test-negative participants than in those infected with SARS-CoV-2 or Influenza A, while fever and headache were significantly more prevalent in the latter, with SARS-CoV-2 cases showing a higher overall symptom burden. These findings support previous reports identifying fever as a key predictor of Influenza A infection [23,28]. Rhinovirus infections, typically confined to the upper respiratory tract in otherwise healthy individuals, were characterized by rhinorrhea and nasal obstruction, consistent with its role as the leading cause of the common cold [23]. Human parainfluenza virus (HPIV), the fourth most prevalent virus in our surveillance, is a comon cause of acute lower respiratory tract infections in children [29]. In our study, HPIV infections were predominantly observed in younger individuals (median age 10 years [IQR 4–27]), contrasting with the older age distribution of test-negative cases (median age 30 years [IQR 15–47]). This pattern mirrors post-pandemic trends reported in Korea and China, where pediatric respiratory viruses—including HPIV, RSV, HCoV, and HBoV, rebounded beyond historical levels following the relaxation of COVID-19 restrictions [27,30]. The resurgence of HPIV in our study likely reflects similar dynamics, with children playing a central role in re-establishing transmission after prolonged low exposure. We also identified a small number of co-infection cases, most commonly involving Influenza A and SARS-CoV-2. The low frequency of co-infections may be explained by viral interference, a phenomenon in which infection by one virus triggers a non-specific innate immune response that inhibits subsequent infections by other viroses [6,15,31]. When comparing clinical presentations between co-infected and mono-infected individuals, no significant differences were observed. This suggests that co-infection does not necessarily exacerbate symptom severity and that hybrid immunity may help mitigate clinical outcomes. Similar findings have been reported for SARS-CoV-2 co-infections in community settings [32]. Together, these observations reflect the ongoing transition of SARS-CoV-2 from pandemic to endemic circulation.

We estimated an Influenza A household SAR of 25% (95% CI: 9.8 – 47.0). This value is similar to estimates from North America and Europe, where SAR among household members were observed to be 10% (95% CI 6.8-14.7) and 38% (95% CI 35.0-41.6), respectively [[33], [34], [35]]. However, when compared with the BA.1.∗ variant, which exhibits a higher household secondary attack rate (SAR) reaching ∼50% [13,36], it highlights the superior transmission of this variant compared to influenza. Consequently, it is plausible that control measures which were not able to arrest transmission of SARS-CoV-2 were able to affect the transmission of influenza, given its lower transmissibility in this setting.

We used innovative diagnostic tools, including multiplex RT-qPCR and metagenomics, to rapidly detect and sequence multiple respiratory pathogens simultaneously, streamlining diagnostic workflows and strengthening surveillance to inform public health policies. Although metagenomic protocols are more commonly applied in recent years and manly in hospital settings, our study demonstrates their implementation within an active surveillance system in an urban informal community. These settings account for nearly 30% of the global population and are often marked by limited health services and restricted diagnostic capacity. In these environments, many respiratory infections are likely to go undetected using conventional methods. Incorporating metagenomics into such contexts enhances the ability to monitor the emergence of new viral variants and recombination events, which is essential for anticipating and preventing outbreaks. This approach is aligned with national genomic surveillance priorities in Brazil, which emphasize the importance of strengthening genomic networks for respiratory viruses. Metagenomics provides a comprehensive characterization of microbial communities, enabling the detection of known pathogens, previously unrecognized viruses, and genetic variants [37,38]. Furthermore, these techniques also help reduce unnecessary antibiotic use by distinguishing between viral and bacterial infections, combating antimicrobial resistance, and enhancing overall public health responses to respiratory illnesses.

We acknowledge limitations of our study. Firstly, it was not possible to complete the metagenomic analysis for all samples. However, we analyzed the majority of samples between November 2021 and March 2022, a period marked by a high number of symptomatic respiratory cases. Additionally, multiplex RT-qPCR was conducted for Influenza A, Influenza B, and RSV throughout the entire year of the study. This testing did not identify any increase in the number of cases during the period when metagenomic analysis was not conducted. Secondly, we acknowledge that a potential limitation of our study is the inclusion of only symptomatic cases, which may underestimate the overall burden of disease by not capturing asymptomatic infections that can also contribute to transmission. However, our focus on symptomatic episodes allowed us to establish a clearer link between viral detection and clinical relevance, strengthening the interpretation of our findings in terms of disease burden and public-health impact. This methodological choice was made to ensure that the metagenomic results corresponded to clinically meaningful events rather than incidental carriage. Thirdly, our assumption that all secondary cases within a household were infected by the primary case analysis was a simplification and did not consider infections acquired outside of the household. In addition, only 10 households had more than one resident included in this analysis; therefore, the SAR had a wide confidence interval and should be interpreted with caution. Finally, although multiple attempts were made, it was not possible to complete all visits during each round.

Future studies should explore how hybrid immunity and immune imprinting shape susceptibility to diverse respiratory viruses, allowing for a more precise quantification of immune responses and their breadth across different viral families. Expanding metagenomic surveillance in resource-limited settings will be critical for detecting emerging pathogens and reducing diagnostic gaps, while pediatric-focused research may clarify the role of children in re-establishing viral transmission. Together, these approaches will strengthen preparedness and support more equitable and effective responses to future respiratory epidemic events.

Our findings illustrate how respiratory viruses regained ecological space following the decline of intense SARS-CoV-2 transmission, highlighting the importance of understanding viral interactions in populations with high exposure and hybrid immunity. Continued surveillance and vaccination remain critical to managing co-circulating respiratory pathogens. Public health efforts to control COVID-19 have likely decreased the transmission of this virus. However, there is also a likelihood of off-season peaks due to reduced transmission of SARS-CoV-2, as observed in the previous Omicron period. Moreover, this reduction could be attributed not only to decreased transmission but also to a reduction in risk perception among the population and a relaxation of individual and collective protective measures. Since SARS-CoV-2 and other viruses share similar respiratory and contact-based transmission routes, the preventive measures implemented for COVID-19 have also proven effective against these viruses. The anticipation of sustained reductions in transmission of influenza and other respiratory viruses during the upcoming season hinges on the continued promotion of adherence to nonpharmaceutical interventions and their prolonged application. Finally, using new diagnostic technologies, as metagenomic, can aid in promptly diagnosing community transmission, thereby facilitating adjustments in policy-making strategies as the SARS-CoV-2 pandemic transitions to an endemic period.

## CRediT authorship contribution statement

**Juan P. Aguilar Ticona:** Writing – original draft, Methodology, Investigation, Formal analysis, Conceptualization. **Luciane Amorim Santos:** Writing – original draft, Methodology, Investigation, Conceptualization. **Xiao Meng:** Writing – original draft, Methodology, Investigation, Conceptualization. **Nivison Nery:** Writing – review & editing, Validation, Investigation. **Mariam O. Fofana:** Writing – review & editing, Methodology, Conceptualization. **Laise de Moraes:** Writing – review & editing, Validation, Investigation. **Icaro Morais Strobel:** Writing – review & editing, Investigation. **Renato Vitoriano:** Writing – review & editing, Investigation. **Marina Silveira Cucco:** Writing – review & editing, Investigation. **Emilia M.M. Andrade Belitardo:** Writing – review & editing, Validation, Investigation, Data curation. **Gowtham Thakku:** Writing – review & editing, Investigation. **Jaqueline S. Cruz:** Writing – review & editing, Investigation. **Angela M. Detweiler:** Writing – review & editing, Investigation. **Norma Neff:** Writing – review & editing, Investigation. **Cristina M. Tato:** Writing – review & editing, Investigation. **Mitermayer G. Reis:** Writing – review & editing, Supervision, Resources, Funding acquisition. **Federico Costa:** Writing – review & editing, Supervision, Conceptualization. **Derek A.T. Cummings:** Writing – review & editing, Supervision, Methodology, Funding acquisition, Conceptualization. **Albert I. Ko:** Writing – review & editing, Supervision, Methodology, Funding acquisition, Conceptualization. **Ricardo Khouri:** Writing – review & editing, Supervision, Methodology, Funding acquisition, Conceptualization.

## Ethics statement

The study was approved by the Ethics Committee of the Institute of Collective Health (approval number 35405320.0.1001.5030), the Institutional Review Boards of the Instituto Gonçalo Moniz, Oswaldo Cruz Foundation (Fiocruz), and the Brazilian National Commission for Ethics in Research (CAAE numbers 45217415.4.0000.0040, 35405320.0.1001.5030, and 59889922.6.0000.0040). Ethical approval was also obtained from the Yale University Human Research Protection Program (protocol number 2000031554).

## Data availability

All accession numbers and related metadata are provided in Supplementary File 2, which contains the complete list of sequences deposited in public repositories.

GISAID accession numbers: EPI_ISL_10101791; EPI_ISL_11026059; EPI_ISL_11026063; EPI_ISL_11026065 - EPI_ISL_11026068; EPI_ISL_11026071 - EPI_ISL_11026073; EPI_ISL_11026076 -EPI_ISL_11026078; EPI_ISL_11026081; EPI_ISL_11026083; EPI_ISL_11026084; EPI_ISL_11026087; EPI_ISL_11026092 - EPI_ISL_11026101; EPI_ISL_11026105; EPI_ISL_11026107; EPI_ISL_11326451; EPI_ISL_11326455; EPI_ISL_11326483; EPI_ISL_11326533; EPI_ISL_11326534; EPI_ISL_11326538; EPI_ISL_11326540; EPI_ISL_11326546; EPI_ISL_11326554; EPI_ISL_11326568; EPI_ISL_11326573; EPI_ISL_11326580; EPI_ISL_11326584; EPI_ISL_13131769; EPI_ISL_13698615; EPI_ISL_13698617 - EPI_ISL_13698620; EPI_ISL_14005455; EPI_ISL_14005459; EPI_ISL_14005463; EPI_ISL_14005464; EPI_ISL_14005468; EPI_ISL_14005471; EPI_ISL_14291104; EPI_ISL_14498245; EPI_ISL_15022953; EPI_ISL_15699040; EPI_ISL_17950794; EPI_ISL_17960445 - EPI_ISL_17960459; EPI_ISL_19561114.

GenBank accession numbers: PQ766149 - PQ766151; PQ779050 - PQ779060; PQ780763; PQ811894 - PQ811897; PQ817249; PQ817250; PQ820482 - PQ820491; PQ834425; PQ834426.

## Funding statement

This work was supported by the 10.13039/100000865Bill & Melinda Gates Foundation (grant number OPP1211988 to M.G.R., F.C. and R.K.); the 10.13039/100000002National Institutes of Health (grant numbers R01 AI121207 to A.I.K., T32AI007517 to M.O.F., R01 AI174105 to A.I.K., F.C. and D.A.T.C.); the 10.13039/501100000265UK Medical Research Council (grant number MR/T029781/1 to F.C.); the 10.13039/100010269Wellcome Trust (grant numbers 102330/Z/13/Z; 218987/Z/19/Z to F.C.); the Conselho Nacional de Desenvolvimento Científico e Tecnológico (Brazilian 10.13039/501100003593National Council for Scientific and Technological Development, 443629/2023-4 and 315470/2021-6 to R.K and PDJ 152748/2024-5 to J.P.A.T.); FIOCRUZ Innovate Program n.02/2023 (postdoctoral fellowship to E.M.M.A.B); the Molecular Surveillance Platform of FIOCRUZ-Bahia and CVSLR/FIOCRUZ (Coordination of 10.13039/100018696Health Surveillance and Reference Laboratories of 10.13039/501100006507Oswaldo Cruz Foundation to R.K.); the Burroughs-10.13039/100004440Wellcome Fund (10.13039/100001949American Society of Tropical Medicine and Hygiene postdoctoral fellowship to M.O.F.); the William H. Prusoff Foundation (postdoctoral fellowship to M.O.F.); the 10.13039/100001547China Medical Board (10.13039/100001547CMB) 10.13039/100006090Global Health Leadership Development Program (Fellowship to M.X.); US 10.13039/100000001National Science Foundation (RAPID award to D.A.T.C.); the Raj and Indra Nooyi Professorship; the Sendas Family; and Beatrice Kleinberg Neuwirth Funds at the 10.13039/100020044Yale School of Public Health (to A.I.K.).

## Declaration of competing interest

The authors declare the following financial interests/personal relationships which may be considered as potential competing interests: A.I.K serves as an expert panel member for Reckitt Global Hygiene Institute, scientific advisory board member for Revelar Biotherapeutics and a consultant for Tata Medical and Diagnostics and Regeneron Pharmaceuticals, and has received grants from Merck, Regeneron Pharmaceuticals and Tata Medical and Diagnostics for research related to COVID-19, all of which are outside the scope of the submitted work. D.A.T.C. has received a grant from Merck and from Pfizer for research unrelated to COVID-19, outside of the scope of this work. D.A.T.C serves on the external advisory board of the Translational Global Infectious Diseases Research Center at the University of Vermont. Other authors declare no competing interests. If there are other authors, they declare that they have no known competing financial interests or personal relationships that could have appeared to influence the work reported in this paper.
