# Supplementary material for: Metagenomic surveillance reveals off-season circulation of respiratory viruses during the COVID-19 pandemic in Salvador, Brazil
Source: New Microbes New Infect. 2026 Feb 6;70:101717. doi: 10.1016/j.nmni.2026.101717 (PMC12925072; doi:10.1016/j.nmni.2026.101717)
Supplement: Multimedia component 1 [file mmc1.docx]

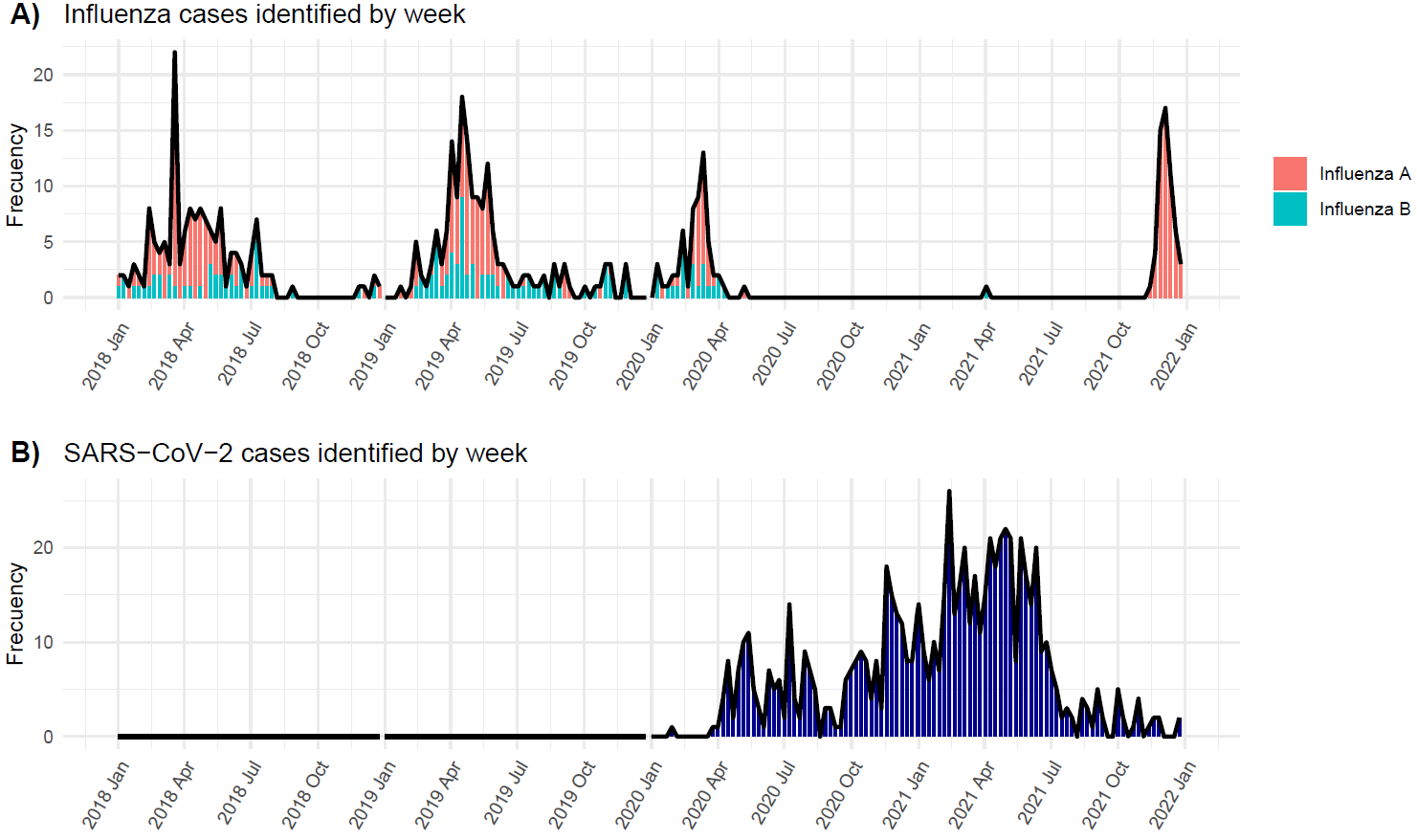


Supplementary Figure 1. Disruption of influenza transmission in Salvador, Brazil. (A) Influenza A and B before and during the COVID-19 pandemic; (B) Emergence of SARS-CoV-2.
