# Supplementary material for: Metagenomic surveillance reveals off-season circulation of respiratory viruses during the COVID-19 pandemic in Salvador, Brazil
Source: New Microbes New Infect. 2026 Feb 6;70:101717. doi: 10.1016/j.nmni.2026.101717 (PMC12925072; doi:10.1016/j.nmni.2026.101717)
Supplement: Multimedia component 2 [file mmc2.docx]

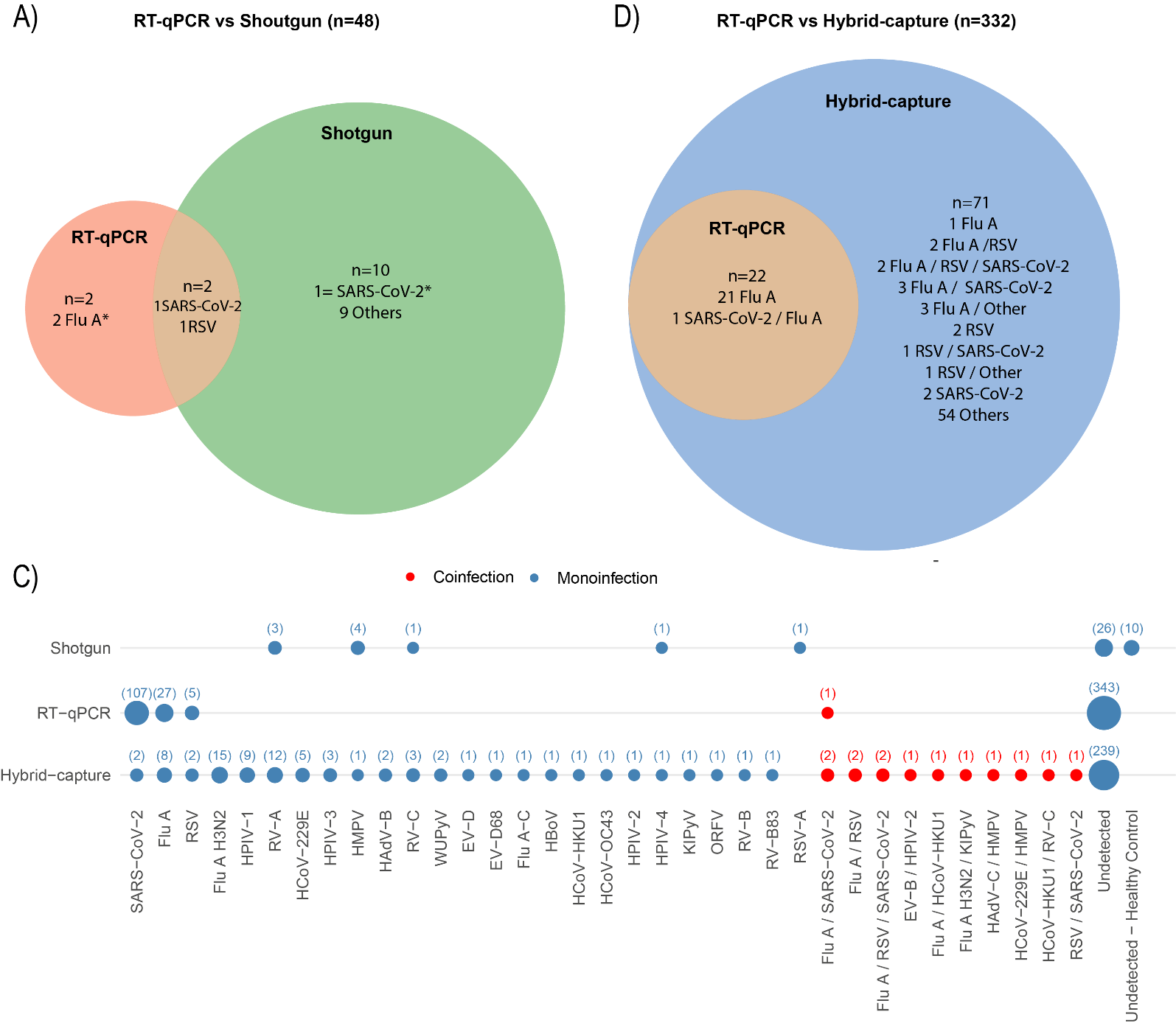


Supplementary Figure 2. Cross-platform Comparison of RT-qPCR, Shotgun Metagenomics, and Hybrid-capture Sequencing for Respiratory Pathogen Detection. A) RT-qPCR versus Shotgun Metagenomics; B) RT-qPCR versus Hybrid-capture Sequencing; and C) Overall pathogen detection frequencies, including coinfections.

* Identified Flu A by PCR and SARS-CoV-2 by Shotgun in the same participants
