# Supplementary material for: Metagenomic surveillance reveals off-season circulation of respiratory viruses during the COVID-19 pandemic in Salvador, Brazil
Source: New Microbes New Infect. 2026 Feb 6;70:101717. doi: 10.1016/j.nmni.2026.101717 (PMC12925072; doi:10.1016/j.nmni.2026.101717)
Supplement: Multimedia component 3 [file mmc3.docx]

Supplementary Table 1: Households and participants visited during the bi-weekly rounds of active case finding

| **Round** | **Date** | **Household (HH) visited** | | **number of residents in the household reported by head of the HH** | |
| --- | --- | --- | --- | --- | --- |
|  |  | **(n=1174)** | | **(n=3364)** | |
| 1 | Nov 10 to Nov 23, 2021 | 757 | 64.48% | 2204 | 65.52% |
| 2 | Nov 24 to Dec 07, 2021 | 904 | 77.00% | 2679 | 79.64% |
| 3 | Dec 08 to Dec 21, 2021 | 938 | 79.90% | 2649 | 78.75% |
| break | Dec 22 to Jan 11 |  | 0.00% |  | 0.00% |
| 4 | Jan 11 to Jan 24, 2022 | 616 | 52.47% | 1828 | 54.34% |
| 5 | Jan 25 to Feb 7, 2022 | 852 | 72.57% | 2462 | 73.19% |
| 6 | Feb 8 to Feb 21, 2022 | 831 | 70.78% | 2438 | 72.47% |
| 7 | Feb 22 to Mar 07, 2022 | 738 | 62.86% | 2142 | 63.67% |
| 8 | Mar 08 to Mar 21, 2022 | 705 | 60.05% | 2027 | 60.26% |
| 9 | Mar 22 to Apr 04, 2022 | 705 | 60.05% | 1990 | 59.16% |
| 10 | Apr 05 to Apr 18, 2022 | 894 | 76.15% | 2585 | 76.84% |
| 11 | Apr 19 to May 2, 2022 | 694 | 59.11% | 2003 | 59.54% |
| 12 | May 3 to May 16, 2022 | 248 | 21.12% | 778 | 23.13% |
| 13 | May 17 to May 30, 2022 | 525 | 44.72% | 1569 | 46.64% |
| 14 | May 31 to June 13, 2022 | 850 | 72.40% | 2447 | 72.74% |
| 15 | June 14 to June 17, 2022 | 397 | 33.82% | 1248 | 37.10% |
| break | June 18 to July 05, 2022 |  | 0.00% |  | 0.00% |
| 16 | July 06 to July 19, 2022 | 879 | 74.87% | 2597 | 77.20% |
| 17 | July 20 to August 2 | 714 | 60.82% | 2150 | 63.91% |
| 18 | August 3 to August 16 | 856 | 72.91% | 2501 | 74.35% |
| 19 | August 17 to August 30 | 961 | 81.86% | 2768 | 82.28% |
| 20 | August 31 to Sep 13 | 960 | 81.77% | 2710 | 80.56% |
| 21 | Sep 14 to Sep 27 | 903 | 76.92% | 2604 | 77.41% |
| 22 | Sep 28 to Oct 11 | 847 | 72.15% | 2446 | 72.71% |
| 23 | Oct 12 to Oct 25 | 839 | 71.47% | 2418 | 71.88% |
| 24 | Oct 26 to Nov 7 | 866 | 73.76% | 2473 | 73.51% |
