# Supplementary material for: Metagenomic surveillance reveals off-season circulation of respiratory viruses during the COVID-19 pandemic in Salvador, Brazil
Source: New Microbes New Infect. 2026 Feb 6;70:101717. doi: 10.1016/j.nmni.2026.101717 (PMC12925072; doi:10.1016/j.nmni.2026.101717)
Supplement: Multimedia component 4 [file mmc4.docx]

Supplementary table 2. Pathogens identified in the metagenomic analysis

| **Full Name** | **Abbreviation** | **Frequency (%)** |
| --- | --- | --- |
| Enterovirus B / Human Parainfluenza Virus 2 | EV-B / HPIV-2 | 1 (0.2%) |
| Enterovirus D | EV-D | 1 (0.2%) |
| Enterovirus D68 | EV-D68 | 1 (0.2%) |
| Influenza A | Flu A | 2 (0.5%) |
| Influenza A-C | Flu A-C | 1 (0.2%) |
| Influenza A / Human Coronavirus HKU1 | Flu A / HCoV-HKU1 | 1 (0.2%) |
| Influenza A / KI Polyomavirus | Flu A / KIPyV | 1 (0.2%) |
| Influenza A / Respiratory Syncytial Virus | Flu A / RSV | 2 (0.5%) |
| Influenza A / SARS-CoV-2 | Flu A / SARS-CoV-2 | 5 (1.2%) |
| Influenza A / SARS-CoV-2 / RSV | Flu A / SARS-CoV-2 / RSV | 2 (0.5%) |
| Human Adenovirus B | HAdV-B | 2 (0.5%) |
| Human Adenovirus C | HAdV-C | 1 (0.2%) |
| Human Adenovirus C / Human Metapneumovirus | HAdV-C / HMPV | 1 (0.2%) |
| Human Bocavirus | HBoV | 1 (0.2%) |
| Human Coronavirus 229E | HCoV-229E | 5 (1.2%) |
| Human Coronavirus 229E / HMPV | HCoV-229E / HMPV | 1 (0.2%) |
| Human Coronavirus HKU1 | HCoV-HKU1 | 1 (0.2%) |
| Human Coronavirus HKU1 / Rhinovirus C | HCoV-HKU1 / RV-C | 1 (0.2%) |
| Human Coronavirus OC43 | HCoV-OC43 | 1 (0.2%) |
| Human Coronavirus OC43 / HCoV-HKU1 | HCoV-OC43 / HCoV-HKU1 | 1 (0.2%) |
| Human Coronavirus OC43 / RSV | HCoV-OC43 / RSV | 1 (0.2%) |
| Human Metapneumovirus | HMPV | 5 (1.2%) |
| Human Parainfluenza Virus 1 | HPIV-1 | 9 (2.2%) |
| Human Parainfluenza Virus 2 | HPIV-2 | 1 (0.2%) |
| Human Parainfluenza Virus 3 | HPIV-3 | 3 (0.7%) |
| Human Parainfluenza Virus 4 | HPIV-4 | 1 (0.2%) |
| KI Polyomavirus | KIPyV | 1 (0.2%) |
| Orf Virus | ORFV | 1 (0.2%) |
| Respiratory Syncytial Virus | RSV | 2 (0.5%) |
| Respiratory Syncytial Virus / SARS-CoV-2 | RSV / SARS-CoV-2 | 1 (0.2%) |
| Rhinovirus A | RV-A | 15 (3.6%) |
| Rhinovirus B | RV-B | 2 (0.5%) |
| Rhinovirus C | RV-C | 4 (1.0%) |
| SARS-CoV-2 | SARS-CoV-2 | 2 (0.5%) |
| WU Polyomavirus | WUPyV | 2 (0.5%) |
| Negative |  | 254 (61.2%) |
| Not tested |  | 82 (19.8%) |
