# Supplementary material for: Metagenomic surveillance reveals off-season circulation of respiratory viruses during the COVID-19 pandemic in Salvador, Brazil
Source: New Microbes New Infect. 2026 Feb 6;70:101717. doi: 10.1016/j.nmni.2026.101717 (PMC12925072; doi:10.1016/j.nmni.2026.101717)
Supplement: Multimedia component 5 [file mmc5.docx]

Table Supplementary Table 3. Frequency of symptoms among participants with coinfections.

| **Variable** | **Overall  (N = 19)** | **Flu A / SARS-CoV-2 (N = 6)** | **Flu A / RSV  (N = 2)** | **Flu A / SARS-CoV-2 / RSV  (N = 2)** | **RSV / SARS-CoV-2  (N = 1)** | **Flu A / HCoV-HKU1  (N = 1)** | **EV-B / HPIV-2  (N = 1)** | **HAdV-C / HMPV (N = 1)** | **HCoV-OC43 / RSV  (N = 1)** | **HCoV-229E / HMPV  (N = 1)** | **HCoV-HKU1 / HCoV-OC43  (N = 1)** | **HCoV-HKU1 / RV-C (N = 1)** | **Flu A H3N2 / KIPyV (N = 1)** |
| --- | --- | --- | --- | --- | --- | --- | --- | --- | --- | --- | --- | --- | --- |
| Age, median (IQR) | 31 (21 - 57) | 38 (31, 68) | 47 (42 - 51) | 41 (21 - 61) | 21 (21 - 21) | 4 (4 - 4) | 4 (4 - 4) | 57 (57 - 57) | 21 (21 - 21) | 5 (5 - 5) | 51 (51 - 51) | 64 (64 - 64) | 28 (28 - 28) |
| Sex, n (%) |  |  |  |  |  |  |  |  |  |  |  |  |  |
| Female | 14 (74%) | 4 (67%) | 2 (100%) | 2 (100%) | 1 (100%) | 0 (0%) | 0 (0%) | 1 (100%) | 0 (0%) | 1 (100%) | 1 (100%) | 1 (100%) | 1 (100%) |
| Male | 5 (26%) | 2 (33%) | 0 (0%) | 0 (0%) | 0 (0%) | 1 (100%) | 1 (100%) | 0 (0%) | 1 (100%) | 0 (0%) | 0 (0%) | 0 (0%) | 0 (0%) |
| Symptoms |  |  |  |  |  |  |  |  |  |  |  |  |  |
| Cough | 17 (89%) | 5 (83%) | 2 (100%) | 2 (100%) | 1 (100%) | 0 (0%) | 1 (100%) | 1 (100%) | 1 (100%) | 1 (100%) | 1 (100%) | 1 (100%) | 1 (100%) |
| Runny nose | 13 (68%) | 3 (50%) | 2 (100%) | 2 (100%) | 1 (100%) | 0 (0%) | 1 (100%) | 0 (0%) | 1 (100%) | 1 (100%) | 1 (100%) | 1 (100%) | 0 (0%) |
| Sore throat | 10 (53%) | 2 (33%) | 2 (100%) | 1 (50%) | 0 (0%) | 0 (0%) | 1 (100%) | 1 (100%) | 0 (0%) | 1 (100%) | 1 (100%) | 1 (100%) | 0 (0%) |
| Shortness of breath | 5 (26%) | 1 (17%) | 1 (50%) | 1 (50%) | 1 (100%) | 0 (0%) | 0 (0%) | 1 (100%) | 0 (0%) | 0 (0%) | 0 (0%) | 0 (0%) | 0 (0%) |
| Fever | 7 (37%) | 1 (17%) | 1 (50%) | 1 (50%) | 0 (0%) | 1 (100%) | 1 (100%) | 0 (0%) | 0 (0%) | 1 (100%) | 0 (0%) | 0 (0%) | 1 (100%) |
| Chills | 0 (0%) | 0 (0%) | 0 (0%) | 0 (0%) | 0 (0%) | 0 (0%) | 0 (0%) | 0 (0%) | 0 (0%) | 0 (0%) | 0 (0%) | 0 (0%) | 0 (0%) |
| Headache | 10 (53%) | 2 (33%) | 2 (100%) | 1 (50%) | 1 (100%) | 1 (100%) | 0 (0%) | 1 (100%) | 1 (100%) | 0 (0%) | 1 (100%) | 0 (0%) | 0 (0%) |
| Loss taste | 3 (16%) | 1 (17%) | 1 (50%) | 0 (0%) | 1 (100%) | 0 (0%) | 0 (0%) | 0 (0%) | 0 (0%) | 0 (0%) | 0 (0%) | 0 (0%) | 0 (0%) |
| Loss smell | 2 (11%) | 1 (17%) | 0 (0%) | 0 (0%) | 1 (100%) | 0 (0%) | 0 (0%) | 0 (0%) | 0 (0%) | 0 (0%) | 0 (0%) | 0 (0%) | 0 (0%) |
| Fatigue | 4 (21%) | 0 (0%) | 2 (100%) | 1 (50%) | 0 (0%) | 0 (0%) | 0 (0%) | 1 (100%) | 0 (0%) | 0 (0%) | 0 (0%) | 0 (0%) | 0 (0%) |
| Myalgia | 4 (21%) | 2 (33%) | 1 (50%) | 0 (0%) | 0 (0%) | 0 (0%) | 0 (0%) | 1 (100%) | 0 (0%) | 0 (0%) | 0 (0%) | 0 (0%) | 0 (0%) |
| Anorexia | 0 (0%) | 0 (0%) | 0 (0%) | 0 (0%) | 0 (0%) | 0 (0%) | 0 (0%) | 0 (0%) | 0 (0%) | 0 (0%) | 0 (0%) | 0 (0%) | 0 (0%) |
| Nausea | 1 (5.3%) | 0 (0%) | 0 (0%) | 0 (0%) | 0 (0%) | 0 (0%) | 0 (0%) | 1 (100%) | 0 (0%) | 0 (0%) | 0 (0%) | 0 (0%) | 0 (0%) |
| Diarrhea | 2 (11%) | 0 (0%) | 2 (100%) | 0 (0%) | 0 (0%) | 0 (0%) | 0 (0%) | 0 (0%) | 0 (0%) | 0 (0%) | 0 (0%) | 0 (0%) | 0 (0%) |
| Altered mental state | 1 (5.3%) | 0 (0%) | 1 (50%) | 0 (0%) | 0 (0%) | 0 (0%) | 0 (0%) | 0 (0%) | 0 (0%) | 0 (0%) | 0 (0%) | 0 (0%) | 0 (0%) |
| No. of symptoms, median (IQR) | 3 (1 - 3) | 1.50 (1.00, 3.00) | 3 (3 - 3) | 3 (2 - 4) | 3 (3 - 3) | 0 (0 - 0) | 3 (3 - 3) | 3 (3 - 3) | 2 (2 - 2) | 3 (3 - 3) | 3 (3 - 3) | 2 (2 - 2) | 1 (1 - 1) |

Flu A, Influenza A virus; RSV, Respiratory Syncytial Virus; SARS-CoV-2, Severe Acute Respiratory Syndrome Coronavirus 2; HCoV, Human Coronavirus (HCoV-229E, HCoV-HKU1, HCoV-OC43); EV-B, Enterovirus B; HPIV-2, Human Parainfluenza Virus type 2; HAdV-C, Human Adenovirus species C; HMPV, Human Metapneumovirus; RV-C, Rhinovirus species C; KIPyV, KI Polyomavirus; IQR, Interquartile Range.
