# Supplementary material for: Metagenomic surveillance reveals off-season circulation of respiratory viruses during the COVID-19 pandemic in Salvador, Brazil
Source: New Microbes New Infect. 2026 Feb 6;70:101717. doi: 10.1016/j.nmni.2026.101717 (PMC12925072; doi:10.1016/j.nmni.2026.101717)
Supplement: Multimedia component 6 [file mmc6.docx]

Supplementary Table 4. Comparison of Influenza A/SARS-CoV-2 coinfection symptoms with Influenza A and SARS-CoV-2 monoinfections.

|  | **Flu A / SARS-CoV-2** | **SARS-CoV-2** | **p-value** | **Flu A / SARS-CoV-2** | **FluA** | **p-value** |
| --- | --- | --- | --- | --- | --- | --- |
|  | N = 7 | N = 109 |  | N = 6 | N = 29 |  |
| Age, median (IQR) | 38.0 (31.0, 68.0) | 36.0 (22.0, 51.0) | 0.424 | 38.0 (31.0, 68.0) | 31.0 (13.0, 38.0) | 0.100 |
| Sex, n (%) |  |  | >0.999 |  |  | >0.999 |
| Female | 4 (66.7%) | 72 (66.1%) |  | 4 (66.7%) | 18 (62.1%) |  |
| Male | 2 (33.3%) | 37 (33.9%) |  | 2 (33.3%) | 11 (37.9%) |  |
| Symptoms |  |  |  |  |  |  |
| Cough | 5 (83.3%) | 84 (77.1%) | >0.999 | 5 (83.3%) | 21 (72.4%) | >0.999 |
| Runny nose | 3 (50.0%) | 70 (64.2%) | 0.667 | 3 (50.0%) | 22 (75.9%) | 0.322 |
| Sore throat | 2 (33.3%) | 61 (56.0%) | 0.407 | 2 (33.3%) | 8 (27.6%) | >0.999 |
| Shortness of breath | 1 (16.7%) | 16 (14.7%) | >0.999 | 1 (16.7%) | 2 (6.90%) | 0.442 |
| Fever | 1 (16.7%) | 57 (52.3%) | 0.114 | 1 (16.7%) | 18 (62.1%) | 0.073 |
| Chills | 0 (0%) | 23 (21.1%) | 0.598 | 0 (0%) | 5 (17.2%) | 0.561 |
| Headache | 2 (33.3%) | 69 (63.3%) | 0.201 | 2 (33.3%) | 23 (79.3%) | 0.043 |
| Loss taste | 1 (16.7%) | 9 (8.26%) | 0.428 | 1 (16.7%) | 4 (13.8%) | >0.999 |
| Loss smell | 1 (16.7%) | 6 (5.50%) | 0.320 | 1 (16.7%) | 2 (6.90%) | 0.442 |
| Fatigue | 0 (0%) | 22 (20.2%) | 0.594 | 0 (0%) | 8 (27.6%) | 0.299 |
| Myalgia | 2 (33.3%) | 25 (22.9%) | 0.624 | 2 (33.3%) | 6 (20.7%) | 0.602 |
| Anorexia | 0 (0%) | 14 (12.8%) | >0.999 | 0 (0%) | 13 (44.8%) | 0.064 |
| Nausea | 0 (0%) | 15 (13.8%) | >0.999 | 0 (0%) | 3 (10.3%) | >0.999 |
| Diarrhea | 0 (0%) | 14 (12.8%) | >0.999 | 0 (0%) | 3 (10.3%) | >0.999 |
| Altered mental state | 0 (0%) | 2 (1.83%) | >0.999 | 0 (0%) | 0 (0%) | >0.1000 |
| No. of symptoms, median (IQR) | 1.5 (1.0, 3.0) | 2.0 (1.0, 3.0) | 0.361 | 1.5 (1.0, 3.0) | 2.0 (1.0, 2.0) | >0.999 |

Flu A, Influenza A virus; SARS-CoV-2, Severe Acute Respiratory Syndrome Coronavirus 2; IQR, Interquartile Range
