# Supplementary material for: Metagenomic surveillance reveals off-season circulation of respiratory viruses during the COVID-19 pandemic in Salvador, Brazil
Source: New Microbes New Infect. 2026 Feb 6;70:101717. doi: 10.1016/j.nmni.2026.101717 (PMC12925072; doi:10.1016/j.nmni.2026.101717)
Supplement: Multimedia component 7 [file mmc7.docx]

Supplementary table 5. SARS-CoV-2 variants identified in the WGS analysis.

| SARS-CoV-2 variants | N = 69 |
| --- | --- |
|  |  |
| AY.43 (Delta) | 2 (2.9%) |
| BA.1 (Omicron) | 28 (41%) |
| BA.1.1 (Omicron) | 23 (33%) |
| BA.1.14 (Omicron) | 1 (1.4%) |
| BA.1.14.1 (Omicron) | 4 (5.8%) |
| BA.1.14.2 (Omicron) | 1 (1.4%) |
| BA.1.9 (Omicron) | 1 (1.4%) |
| BA.2 (Omicron) | 1 (1.4%) |
| BA.2.12.1 (Omicron) | 1 (1.4%) |
| BA.4 (Omicron) | 1 (1.4%) |
| BA.5.1 (Omicron) | 3 (4.3%) |
| BA.5.2.1 (Omicron) | 2 (2.9%) |
| BE.10 (Omicron) | 1 (1.4%) |
