# Supplementary material for: Metagenomic surveillance reveals off-season circulation of respiratory viruses during the COVID-19 pandemic in Salvador, Brazil
Source: New Microbes New Infect. 2026 Feb 6;70:101717. doi: 10.1016/j.nmni.2026.101717 (PMC12925072; doi:10.1016/j.nmni.2026.101717)
Supplement: Multimedia component 8 [file mmc8.docx]

Supplementary table 6. Symptoms associated with respiratory virus infections, evaluated using odds ratio estimates.

|  | SARS-CoV-2 | | **FluA** | | **Rinovirus** | | **HPIV** | | **RSV** | |
| --- | --- | --- | --- | --- | --- | --- | --- | --- | --- | --- |
|  | **OR** | **95% CI** | **OR** | **95% CI** | **OR** | **95% CI** | **OR** | **95% CI** | **OR** | **95% CI** |
| Age | 1.01 | 1.00, 1.02 | 0.99 | 0.97, 1.01 | 0.99 | 0.96, 1.01 | 0.96 | 0.92, 0.99 | 1.00 | 0.96, 1.03 |
| Sex |  |  |  |  |  |  |  |  |  |  |
| Female | — | — | — | — | — | — | — | — | — | — |
| Male | 0.92 | 0.58, 1.44 | 1.09 | 0.48, 2.35 | 1.10 | 0.42, 2.68 | 0.99 | 0.30, 2.94 | 0.71 | 0.10, 3.37 |
| Cough |  |  |  |  |  |  |  |  |  |  |
| No | — | — | — | — | — | — | — | — | — | — |
| Yes | 0.41 | 0.24, 0.73 | 0.32 | 0.14, 0.82 | 0.74 | 0.24, 3.26 | 0.74 | 0.19, 4.87 | 0.16 | 0.03, 0.86 |
| Runny nose |  |  |  |  |  |  |  |  |  |  |
| No | — | — | — | — | — | — | — | — | — | — |
| Yes | 1.06 | 0.68, 1.67 | 1.85 | 0.81, 4.80 | 3.54 | 1.17, 15.3 | 1.06 | 0.36, 3.52 | 1.47 | 0.31, 10.4 |
| Sore throat |  |  |  |  |  |  |  |  |  |  |
| No | — | — | — | — | — | — | — | — | — | — |
| Yes | 1.88 | 1.22, 2.92 | 0.56 | 0.23, 1.26 | 1.34 | 0.55, 3.27 | 1.97 | 0.67, 6.10 | 0.25 | 0.01, 1.46 |
| Shortness of breath |  |  |  |  |  |  |  |  |  |  |
| No | — | — | — | — | — | — | — | — | — | — |
| Yes | 0.99 | 0.52, 1.78 | 0.43 | 0.07, 1.48 | 1.35 | 0.38, 3.83 | 0.96 | 0.15, 3.65 | 0.96 | 0.05, 5.76 |
| Fever |  |  |  |  |  |  |  |  |  |  |
| No | — | — | — | — | — | — | — | — | — | — |
| Yes | 1.98 | 1.28, 3.08 | 2.96 | 1.37, 6.66 | 0.72 | 0.25, 1.83 | 1.36 | 0.44, 3.99 | 0.00 |  |
| Chills |  |  |  |  |  |  |  |  |  |  |
| No | — | — | — | — | — | — | — | — | — | — |
| Yes | 1.78 | 1.01, 3.09 | 1.39 | 0.45, 3.56 | 0.70 | 0.11, 2.53 | 1.11 | 0.17, 4.25 | 0.00 |  |
| Headache |  |  |  |  |  |  |  |  |  |  |
| No | — | — | — | — | — | — | — | — | — | — |
| Yes | 2.10 | 1.35, 3.30 | 4.67 | 1.97, 12.9 | 0.91 | 0.36, 2.21 | 3.04 | 1.00, 11.3 | 0.20 | 0.01, 1.20 |
| Loss taste |  |  |  |  |  |  |  |  |  |  |
| No | — | — | — | — | — | — | — | — | — | — |
| Yes | 1.08 | 0.46, 2.29 | 1.91 | 0.53, 5.41 | 1.99 | 0.45, 6.39 | 0.00 |  | 0.00 |  |
| Loss smell |  |  |  |  |  |  |  |  |  |  |
| No | — | — | — | — | — | — | — | — | — | — |
| Yes | 0.76 | 0.28, 1.80 | 0.97 | 0.15, 3.50 | 3.07 | 0.83, 9.10 | 0.00 |  | 0.00 |  |
| Fatigue |  |  |  |  |  |  |  |  |  |  |
| No | — | — | — | — | — | — | — | — | — | — |
| Yes | 1.49 | 0.84, 2.57 | 2.24 | 0.89, 5.17 | 0.62 | 0.10, 2.22 | 0.98 | 0.15, 3.74 | 0.98 | 0.05, 5.91 |
| Myalgia |  |  |  |  |  |  |  |  |  |  |
| No | — | — | — | — | — | — | — | — | — | — |
| Yes | 1.84 | 1.06, 3.14 | 1.61 | 0.57, 3.94 | 1.03 | 0.23, 3.19 | 0.47 | 0.03, 2.47 | 0.00 |  |
| Anorexia |  |  |  |  |  |  |  |  |  |  |
| No | — | — | — | — | — | — | — | — | — | — |
| Yes | 1.09 | 0.55, 2.05 | 6.03 | 2.67, 13.5 | 0.78 | 0.12, 2.83 | 2.97 | 0.79, 9.35 | 0.00 |  |
| Nausea |  |  |  |  |  |  |  |  |  |  |
| No | — | — | — | — | — | — | — | — | — | — |
| Yes | 1.06 | 0.55, 1.96 | 0.77 | 0.18, 2.30 | 0.33 | 0.02, 1.66 | 2.66 | 0.71, 8.35 | 0.00 |  |
| Diarrhea |  |  |  |  |  |  |  |  |  |  |
| No | — | — | — | — | — | — | — | — | — | — |
| Yes | 1.16 | 0.58, 2.19 | 0.91 | 0.21, 2.74 | 1.31 | 0.30, 4.10 | 2.15 | 0.47, 7.24 | 0.00 |  |
| Altered mental state |  |  |  |  |  |  |  |  |  |  |
| No | — | — | — | — | — | — | — | — | — | — |
| Yes | 3.13 | 0.37, 26.3 | 0.00 |  | 0.00 |  | 0.00 |  | 0.00 |  |
| No. of symptoms, median (IQR) | 1.22 | 0.97, 1.53 | 0.97 | 0.64, 1.46 | 1.66 | 1.02, 2.78 | 10.6 | 4.83, 30.7 | 0.89 | 0.39, 2.01 |

Flu A, Influenza A virus; SARS-CoV-2, Severe Acute Respiratory Syndrome Coronavirus 2;
