# Supplementary material for: Metagenomic surveillance reveals off-season circulation of respiratory viruses during the COVID-19 pandemic in Salvador, Brazil
Source: New Microbes New Infect. 2026 Feb 6;70:101717. doi: 10.1016/j.nmni.2026.101717 (PMC12925072; doi:10.1016/j.nmni.2026.101717)
Supplement: Multimedia component 9 [file mmc9.docx]

Supplementary table 7. Multivariable analysis of symptoms associated with respiratory virus infections.

| **SARS-CoV-2** | | | |
| --- | --- | --- | --- |
|  | Odds Ratios | CI | p |
| Cough | 0.26 | 0.13 – 0.53 | **<0.001** |
| Fever | 1.57 | 0.98 – 2.50 | 0.059 |
| Headache | 1.67 | 1.04 – 2.68 | **0.033** |
| No RspSint | 1.52 | 1.16 – 2.01 | **0.002** |
| **FluA** | | | |
|  | Odds Ratios | CI | p |
| Cough | 0.29 | 0.11 – 0.77 | **0.010** |
| Headache | 3.59 | 1.44 – 10.24 | **0.009** |
| Anorexia | 4.76 | 2.03 – 11.09 | **<0.001** |
| **HPIV** | | | |
|  | Odds Ratios | CI | p |
| Runny nose | 3.44 | 1.13 – 14.91 | 0.052 |
| Loss smell | 2.90 | 0.78 – 8.72 | 0.077 |
| **Rinovirus** | | | |
|  | Odds Ratios | CI | p |
| Age | 0.96 | 0.91 – 1.00 | 0.079 |
| No RspSint | 10.15 | 4.54 – 30.83 | **<0.001** |
| **RSV** | | | |
|  | Odds Ratios | CI | p |
| Cough | 0.16 | 0.03 – 0.86 | **0.021** |

Flu A, Influenza A virus; SARS-CoV-2, Severe Acute Respiratory Syndrome Coronavirus 2; HPIV, Human parainfluenza virus, RSV, Respiratory syncytial virus
